# Writing scientific manuscripts: most common mistakes

**DOI:** 10.1590/2177-6709.22.5.113-117.sar

**Published:** 2017

**Authors:** Jorge Faber

**Affiliations:** 1 Universidade de Brasília, Programa de Pós-graduação em Odontologia (Brasília/DF, Brazil).

**Keywords:** Publication, Manuscript, Article, Submission

## Abstract

I have had the privilege of serving as editor-in-chief for 11 years of two scientific journals: The Dental Press Journal of Orthodontics and the Journal of the World Federation of Orthodontists. I had the opportunity to read and correct thousands of manuscripts. This experience was greatly enriching, because reading a text professionally completely differs from the perspective of readers in general. The routine practice of correcting manuscripts has made me realize that some errors recur frequently. To help authors to improve their manuscripts before submission, these problems are discussed here in the order that they appear in conventional manuscript sections.

## RESUMO

Ao longo dos 11 anos em que atuei como editor-chefe de revistas científicas (*Dental Press Journal of Orthodontics* e *Journal of the World Federation of Orthodontists*), tive a oportunidade de ler e corrigir milhares de manuscritos - manuscrito, no jargão editorial, é o trabalho submetido para publicação ainda não editado e publicado. Essa experiência foi muito enriquecedora, pois a leitura profissional de um texto difere totalmente daquela realizada pelos leitores em geral. O lidar corriqueiro com as correções de manuscritos me fez perceber que alguns erros são muito recorrentes. Eles serão discutidos na ordem das seções apresentadas nos trabalhos, com o objetivo de ajudar os autores a melhorar seus manuscritos antes da submissão.

## INTRODUCTION

Authors often underestimate the Introduction, the first section of a manuscript, in both its relevance and its complexity. The relative rigidity and concision required in scientific texts should not eclipse the elegance of a beautifully written text. However, these attributes pose very specific challenges to authors. I see these difficulties materialized into the same errors repeatedly in submitted manuscripts.

The most common mistake is to write a too long Introduction.^1^ This may be justified by several reasons, but is often the result of the fact that many manuscripts originate from dissertations or theses in which reviews of the literature might be relatively long. After great efforts to write beautiful reviews of the literature, some authors tend to cling to the quality of their production and want to share it with other readers. The major problem here is that most of us are not interested in long, non-objective texts. There is no specific size limit of the Introduction, but a rule of thumb is to limit the word count to about 10% of the total number of words in the manuscript.

The second most common error is lack of coherence.^1^ Sometimes studies focus on many different question, and their rationale is unclear. The Introduction often begins with a paragraph that contextualizes the theme of the study and presents the state of the art of what is under analysis. Authors should gradually guide the reader’s thoughts to the objectives of the study, which are always described in the last paragraph of the Introduction. However, ideas should be organized so that, immediately before reading the objective, the reader understands the relevance of the topic and anticipates which gap in knowledge has to be filled.

The number of references should be limited to what is actually necessary. The most innovative studies tend to list few references, and an excessively large number of quotes has a negative effect on the most qualified readers, as it suggests that the study does not bring anything new to the literature, or that references were included without following any criterion. When using references to other studies, we should avoid using the name of authors in the text or, especially, as the subject of sentences.^1^

For example, instead of:

*Kim,^10^ when analyzing the prevalence of anterior crossbite in 1897 children with complete primary dentition, detected that, during the period of primary dentition, the factors for the incidence of this type of malocclusion were 43.6% genetic and 56.4% postnatal.*

Use:

*The factors of incidence of anterior crossbite in children with complete primary dentition are genetic in 56.4% of the cases and postnatal in 43.6%.*

Different writing styles highlight different aspects. While in the first example, the main element of the sentence is the author, in the second, the information provided gains prominence. Older manuscripts used to mention numerous names of authors, and this remains a current practice in philosophical fields, such as in Law. This stylistic change along time may be partly assigned to the loss of relevance of the argument of the authority and the growing importance level of evidence. Today, it does not matter who the author of a sentence or idea is. The important element is the level of evidence provided by the source. That does not mean that no author names should be mentioned, but this should be the exception, not the rule, and in general only to acknowledge the great importance of a publication for that specific study.

These are the most common shortcomings when writing the Introduction section. When there is any question about how to approach what and when, remember the KISS acronym: Keep It Simple, Scientist.

### Guidelines

1. Be concise: no one wants to read excessively long studies.
2. As a rule, the Introduction should not be longer than 10% of the total length of the manuscript.
3. Pay special attention to text coherence and cohesion.
4. Do not present long reviews of the literature; use the literature to set the context for the problem under study.
5. Avoid sentences in which the authors of articles are the subject.

## MATERIAL AND METHODS

The Material and Methods (MM) section often has errors that originate in the nature of its own construction. It is written at several phases of the study and at different points of its generation. Therefore, writing atavisms are frequent. To make myself clearer: the MM section is first written as part of a project. At that point, the final study design has not been fully established and, consequently, the verb tense should be the simple future. At the time when the study becomes a manuscript for publication, the verb tense should be changed to the simple past. All the MM section should be written in the past tense, because methods refer to what has been done, not to something that will be or is currently being carried out.

A recurrent problem in several manuscripts that never get to the pages of a scientific journal is the lack of approval by an institutional review board (IRB) or ethics in research committee. Ideally, the MM section should include in the first paragraph the information that this approval was been obtained. Although several aspiring authors may see this approval as a merely bureaucratic requirement, the analysis by an IRB provides important protection to the individuals and animals that are, somehow, part of the study. These committees do not often grant approval after the study has been conducted, that is, if the author has not submitted for approval before the study started, it is very likely that approval will be refused, and the study results might never be published.

Incomplete data are also frequent. Lack of information often results from the fact that authors have such a profound knowledge of their investigations that no information left out will affect their manuscript comprehension. However, such gap will definitely affect its understanding by other readers. Such inconsistencies are also frequent because the original project undergoes reviews, and some materials and steps are changed.

Additionally, authors often submit incomplete descriptions of their studies, which has a negative impact on its reproducibility. A scientific study must always be reproducible. It should include information about the materials used, such as the active agents, manufacturers and place of manufacture.

Sometimes materials are described in a way that makes the manuscript read as an advertisement. Authors should use writing styles that distance themselves from endorsing techniques or materials used.

Finally, a very common error is not to include a detailed description of statistical methods. Such description should be at the end of the MM section. Several factors may explain this absence. The most important may be that most authors have a limited knowledge of statistics, which complicates the preparation of this manuscript section. The statistical methods are described only many months after statisticians have conducted the analyses, and this temporal gap may negatively affect descriptions.

Some study methods are very complex, and, whenever possible, authors should ask an external reviewer to read the MM section and revise it before submitting the manuscript to a journal.

### Guidelines

1. Write all the section in the past tense.
2. Never forget to include IRB approval.
3. Describe all methods thoroughly.
4. Include all the materials used, as well as information about their manufacturers.
5. When conducting the statistical analysis of your study data, ask the statistician to describe all methods as they should be published. Do not fail to include a detailed description of those methods in your manuscript.

## RESULTS

The Results section is often inadequately short. Some authors may summarize findings insufficiently and then only refer tables and graphs. Paradoxically, authors are also often verbose and show data in tables and graphs that repeat what has been described in the text. Tables are usually great means of showing results. However, authors have to be familiar with how to organize data in tables. A useful tip is to check how other authors have shown their results and get inspiration to prepare your own findings.

An interesting format for the presentation of results is to write about the most important points in the text and then refer to graphs and tables that show findings in details.

**Figure 1:**
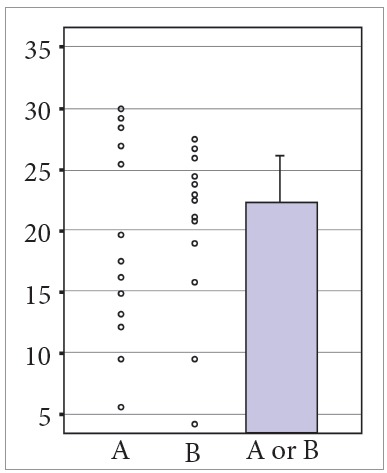
Example of bar and line graph. This type of graph shows different data distributions (A or B) in exactly the same visual representation, which makes it impossible for the readers to examine data accurately. Therefore, although often found in scientific manuscripts, line and bar graphs should be avoided at all times.

Tables are usually richer than graphs, but graphs may be a good tool to show results. However, some graphic presentations should be avoided whenever possible, such as, and especially, bar and line graphs^3^. Figures in scientific communications are extremely relevant because they visually and intuitively show data that otherwise would have to be read. Some images are worth more than words, and this resource should be used wisely and creatively in scientific manuscripts. However, bar graphs are seriously limited when data have to be detailed. In this type of graph, different distributions may have exactly the same graphic distribution (Fig 1). An alternative is boxplots, as they clearly show the distribution of data and use visual resources to present results.

Another difficulty in being accepted for publication is the poor quality of illustrations in general. The most representative examples of this in Orthodontics are low-quality cephalometric tracings, prepared on white paper using felt tip pens. Such tracings do not capture the patient's anatomy, but, despite that, they are often submitted to orthodontic journals. They are imprecise and hastily prepared. Other examples are photos, either clinical or of study methods, that are obtained using cell phones, which do not have standardized focal distances or lighting parameters to take photos with the quality required for publication. When material is submitted like this, editors, reviewers and readers may justly wonder whether authors that were unable to carefully prepare their illustrations may have been sufficiently careful about conducting their research. Numerous manuscripts are rejected because of photos and figures.

### Guidelines

1. Do not be too concise.
2. Avoid being verbose. Briefly report most important findings and then refer tables and graphs.
3. Avoid bar and line graphs.
4. Include professional quality illustrations.

## DISCUSSION

The Discussion is the heart of all scientific studies and the section where the authors should express their interpretative creativity and capacity. Several cases of relevant scientific results have gone unnoticed by the scientific community because their authors failed to interpret results. This means that data should be interpreted. Authors do not have to follow other authors’ claims to argue in favor of an idea. This section is where the authors may be bold, make propositions and suggestions, and explain results; in other words, this is where they may introduce innovative interpretations. At the same time, this is where criticism to other studies that have noteworthy flaws should be made.

The most common error in this section is to write it as a literature review. The Discussion section should not be a review of the literature; it should compare and contrast findings with those reported by other authors and explain their differences and similarities. 

Another frequent shortcoming is failing to include a presentation of the study limitations. Honesty in clearly presenting limitations shows that the authors analyzed their study comprehensively. Failing to include limitations may convey the idea - often correct - that the authors simply did not understand the exact scope of the study that they have conducted.

Finally, any published study has to deal with all the results presented in the Discussion section. As a rule, if a set of data was presented, it must be discussed. Not included in this rule are minor details, such as data distribution normality and error of the method, which are discussed only when they have such relevant impact on data that they deserve specific consideration.

### Guidelines

1. Do not make a review of the literature: use the literature to compare your results with those of other studies.
2. Make clear what the study limitations are.
3. All results reported should be fully discussed in the manuscript.

## CONCLUSION

The Conclusion section should be simple. The most common problem in this section is not addressing all the objectives listed in the beginning of the study.

The second most common problem is the presentation of conclusions that are beyond the scope of the study design; for example, a case series that discusses the evaluation of extra-radicular mini implants. During the retraction of mandibular teeth to correct Class III malocclusion, conclusions should not include, for example, that extra-radicular implants are better than extraction treatments, as the study has not investigated that.

## FINAL CONSIDERATIONS

The purpose of this article is to improve the quality of manuscripts that authors submit to scientific journals. Following the suggestions described above will increase the chances of acceptance for publication. However, authors should be aware that writing a scientific manuscript demands careful attention and many hours of work. Even experienced authors write and review their manuscripts several times before submitting them to a journal. In science, as in literature, an author’s reputation is not shaped by the number of publications, but by the quality of what is produced.
